# Transmission of SARS-CoV-2 Involving Residents Receiving Dialysis in a Nursing Home — Maryland, April 2020

**DOI:** 10.15585/mmwr.mm6932e4

**Published:** 2020-08-14

**Authors:** Benjamin F. Bigelow, Olive Tang, Gregory R. Toci, Norberth Stracker, Fatima Sheikh, Kara M. Jacobs Slifka, Shannon A. Novosad, John A. Jernigan, Sujan C. Reddy, Morgan J. Katz

**Affiliations:** ^1^Department of Medicine, Johns Hopkins University School of Medicine, Baltimore, Maryland; ^2^Division of Population Health and Disease Prevention, Baltimore City Health Department, Baltimore, Maryland; ^3^CDC COVID-19 Response Team.

SARS-CoV-2, the virus that causes coronavirus disease 2019 (COVID-19), can spread rapidly in nursing homes once it is introduced (*1*,*2*). To prevent outbreaks, more data are needed to identify sources of introduction and means of transmission within nursing homes. Nursing home residents who receive hemodialysis (dialysis) might be at higher risk for SARS-CoV-2 infections because of their frequent exposures outside the nursing home to both community dialysis patients and staff members at dialysis centers (*3*). Investigation of a COVID-19 outbreak in a Maryland nursing home (facility A) identified a higher prevalence of infection among residents undergoing dialysis (47%; 15 of 32) than among those not receiving dialysis (16%; 22 of 138) (p<0.001). Among residents with COVID-19, the 30-day hospitalization rate among those receiving dialysis (53%) was higher than that among residents not receiving dialysis (18%) (p = 0.03); the proportion of dialysis patients who died was 40% compared with those who did not receive dialysis (27%) (p = 0.42).Careful consideration of infection control practices throughout the dialysis process (e.g., transportation, time spent in waiting areas, spacing of machines, and cohorting), clear communication between nursing homes and dialysis centers, and coordination of testing practices between these sites are critical to preventing COVID-19 outbreaks in this medically vulnerable population.

In April 2020, a COVID-19 outbreak occurred at a Maryland nursing home (facility A), a 200-bed skilled nursing facility specializing in postacute and long-term care, with an independently operated dialysis center co-located on site. In Maryland, during the month of April, approximately 25% of all SARS-CoV-2 tests had positive results when considering the rolling 7-day average, and approximately half of nursing homes in the state had active outbreaks.^†^ The Maryland Department of Health conducted SARS-CoV-2 testing for symptomatic nursing home residents with a 3–5-day turnaround time for results. Because of the evolving outbreak and limited testing capacity at the health department, a Johns Hopkins response team provided SARS-CoV-2 testing with a 24-hour turnaround time for all facility A residents who had not previously had a positive test result within the past 48 hours. On April 30, SARS-CoV-2 testing was conducted among all facility A residents, and the prevalences among patients receiving and those not receiving dialysis and by floor of residence in facility A were assessed. All statistical analyses were performed using chi-square tests (p<0.05) with Stata statistical software (version 16; StataCorp, LLC).

## Investigation and Findings

On April 16, 2020, the facility census was 170; 75% of residents resided in double-occupancy rooms. Thirty-two (19%) residents were receiving dialysis at the co-located dialysis center. The two schedules for dialysis were Monday, Wednesday, and Friday or Tuesday, Thursday, and Saturday, with three 4-hour shifts per schedule. Shifts overlapped appointment times and residents remained in a dialysis waiting room until their appointment. Facility A residents accounted for 40% of dialysis patients at the center; other patients were from the surrounding community and were scheduled simultaneously with facility A residents.

By April 1, per an order by the Maryland Governor, facility A and the dialysis center required universal surgical masks for all staff members, cancelled group activities and group dining, and prohibited visitors. Staff members were screened for symptoms (e.g., shortness of breath, cough, fever, myalgias, headache, diarrhea, and loss of taste or smell) and their temperature was measured before each shift and being permitted to work. Residents of the nursing home were screened every 8 hours; community dialysis patients were screened before their dialysis appointment.

**FIGURE 1:**
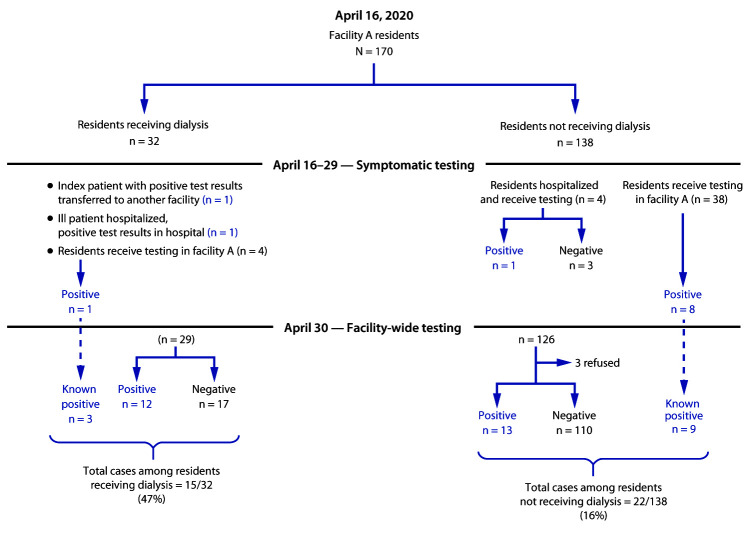
SARS-CoV-2 testing results among residents of a nursing home receiving or not receiving dialysis — Maryland, April 2020

On April 16, a resident at facility A developed an elevated temperature and malaise and subsequently had a positive result for SARS-CoV-2 RNA by reverse transcription–polymerase chain reaction (RT-PCR) testing of a nasopharyngeal swab specimen. This resident (the index patient) received dialysis on the Tuesday, Thursday, and Saturday schedule during shift 2. The patient received dialysis on April 18, and after receiving a positive test result, was transferred to a designated COVID-19 area in another nursing home (Figure 1).

During the following week (beginning April 20), the Maryland Department of Health tested 47 symptomatic residents of the nursing home and 10 symptomatic staff members (symptoms were defined as any of the following: fever >99°F [37.2°C], cough, malaise, headache, or upper respiratory symptoms); 11 residents and three staff members had positive test results for SARS-CoV-2. Two of the infected residents received dialysis on the Monday, Wednesday, and Friday schedule, one each on shifts 2 and 3. All staff members with positive test results were excluded from work.

On April 21, the dialysis center attempted to cohort all residents into four groups: 1) patients with confirmed COVID-19; 2) symptomatic persons with SARS-CoV-2 test results pending; 3) potentially exposed but asymptomatic persons; and 4) asymptomatic, nonexposed persons. Per cohorting strategy, when possible, these groups received dialysis during different shifts; however, because of scheduling constraints, groups 1 and 2 could receive dialysis on the same shift, as could groups 3 and 4. Universal masking was strongly recommended for patients in the dialysis center; however, the center reported patients often had difficulty wearing masks for the entire session. Dialysis center staff members caring for patients with COVID-19 were required to wear gowns, masks, gloves, and eye protection. Efforts were made to separate dialysis machines by 6 feet (2 meters), but because of space limitations, this was not always possible.

On April 30, among the facility’s 164 residents, 152 (93%) had nasopharyngeal specimens tested for SARS-CoV-2 with RT-PCR; three residents refused testing, and nine had previously received positive SARS-CoV-2 test results. Symptom status at the time of universal testing was recorded based on discussion with facility staff members. Among the 152 residents who received testing, 25 (16%) additional SARS-CoV-2 infections were identified, including in 12 (41%) of the 29 remaining residents who were receiving dialysis and in 13 (11%) of the 123 remaining residents who were not receiving dialysis. Among the 25 newly identified cases, 18 (72%) persons were asymptomatic at the time of testing, including seven of 12 and 11 of 13 residents who did and did not receive dialysis, respectively. Two dialysis technicians subsequently became symptomatic, received positive test results (May 1 and May 4) and self-isolated at home. Overall, 40 COVID-19 cases were identified in facility A in 37 residents and three staff members.

**FIGURE 2:**
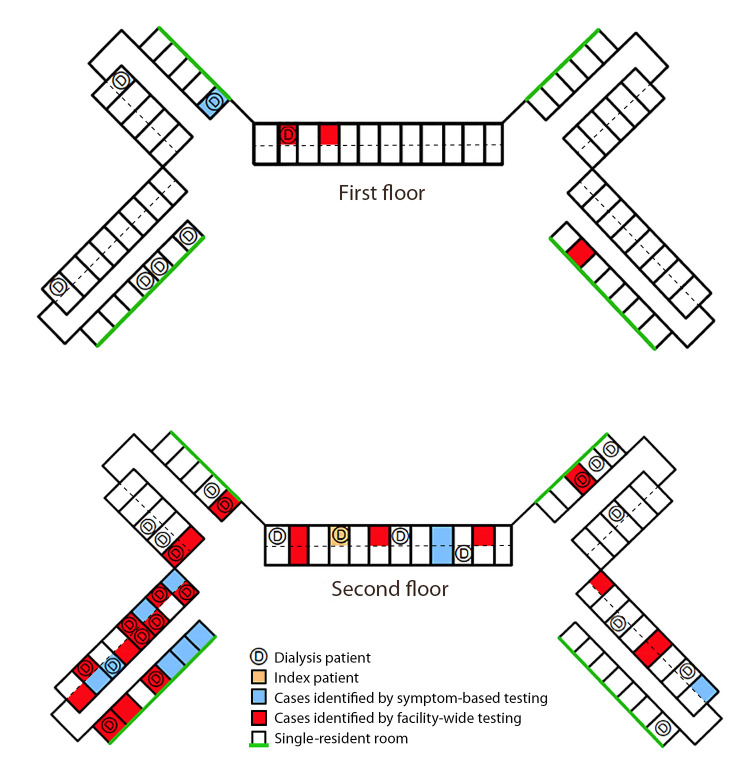
Distribution of COVID-19 cases among facility A residents receiving or not receiving dialysis, by floor* — Maryland, April 2020 **Abbreviations:** COVID-19 = coronavirus disease 2019; D = room of resident receiving dialysis. * All dialysis treatments were completed in the dialysis center, which was co-located on site. Symptom-based testing referred to targeted testing of residents who were experiencing at least one of the following symptoms: fever >99°F (37.2°C), cough, malaise, headache, or upper respiratory symptoms. Facility-wide testing refers to the testing of all facility A residents who had not previously had test results positive for SARS-CoV-2, regardless of symptoms.

As of April 30, 15 of 32 (47%) residents receiving dialysis had positive test results, compared with 22 of 138 (16%) who did not receive dialysis (p<0.001, chi-squared test) (Table). The prevalence of SARS-CoV-2 infection among residents on the second floor of facility A (33 of 81; 41%) was significantly higher than that among residents on the first floor (four of 89; 4.5%) (p = <0.001) (Figure 2).

**TABLE:** Number of residents who had positive test results for SARS-CoV-2 RNA among facility A residents (N = 170), overall and by residence floor and dialysis schedule — Maryland, April 16–30, 2020

Characteristic	No. of residents	No. (%) of cases
**Dialysis status, all residents**		
Not receiving dialysis	138	22 (16)
Receiving dialysis	32	15 (47)
**Facility residence (residents receiving dialysis only)**		
First floor	7	2 (29)
Second floor	25	13 (52)
**Dialysis schedule**		
Monday/Wednesday/Friday	19	9 (47)
Shift 1	4	0 (0)
Shift 2	3	1 (33)
Shift 3	12	8 (67)
Tuesday/Thursday/Saturday	13	6 (46)
Shift 1	6	3 (50)
Shift 2	6	2 (33)
Shift 3	1	1 (100)

Among residents with SARS-CoV-2 infection, those receiving dialysis were more often hospitalized within 30 days of receiving a positive test result (eight of 15) compared with those not receiving dialysis (four of 22; 18%) (p = 0.03). Among residents with SARS-CoV-2 infection, six of 15 residents receiving dialysis and six of 22 (27%) residents not receiving dialysis died within 30 days of diagnosis (p = 0.42). Information on cause of death or comorbidities was not available for residents who died.

## Public Health Interventions

Facility A closed to new admissions after the first case was identified on April 16 and did not accept new admissions until May 8. Testing for symptomatic residents and staff members was conducted during April 16––29. Follow-up facility-wide testing for all residents who had not previously had test results positive for SARS-CoV-2, regardless of symptoms, was conducted on April 30; however, because of testing limitations, asymptomatic staff members and community dialysis patients were not tested. To mitigate transmission among residents, following guidance from the local health department, the facility cohorted residents by test results. All residents, regardless of COVID-19 status, were isolated in their rooms while the facility remained in active outbreak status. Staff members were required to wear personal protective equipment for care of all residents with positive test results and those under observation. Residents with SARS-CoV-2 infection receiving dialysis were scheduled separately from residents who had negative test results.

## Discussion

During a COVID-19 outbreak investigation at a skilled nursing facility in Maryland, testing identified infections in both residents who were and were not receiving dialysis, but disease prevalence was significantly higher among residents receiving dialysis and among residents on the second floor compared with those not receiving dialysis and those on the first floor.

Residents leaving their rooms for dialysis could be a potential source of SARS-CoV-2 introduction into the nursing home and might pose an underrecognized source of transmission, both in the dialysis center and in the nursing home. Better monitoring and understanding of the risks associated with residents who regularly leave the facility for outpatient health care is needed. Implementing procedures that ensure use of masks, social distancing, and improved ventilation during transportation and in waiting areas is important for preventing SARS-CoV-2 transmission.

Nursing home residents who undergo dialysis are a particularly vulnerable population (*3*,*4*). Compared with other residents, they often have more underlying medical conditions, many of which have been associated with more severe SARS-CoV-2 infections, including diabetes mellitus, hypertension, and heart disease (*5*,*6*). This population might also be more frequently exposed to persons outside the nursing home, including community dialysis patients and dialysis center staff members.

Identifying the definitive source for this outbreak or tracing the chain of subsequent transmission was not possible. For example, many residents receiving dialysis were housed on the second floor of the nursing home, and transmission might have occurred within the nursing home, at the dialysis center, or during transportation between the two locations (e.g., in the closed confines of an elevator). Given that shifts overlapped appointment times at the dialysis center, before their dialysis appointments residents might spend time in a waiting area where additional exposures might occur. Further, whereas the first identified cases occurred among residents who were receiving dialysis, given the COVID-19 incubation period of up to 14 days and delayed testing among other residents and staff members, the definitive source of introduction remains unclear. The prevalence of asymptomatic infections poses additional challenges to identifying the source of introduction and tracing transmission through the facility (*7*,*8*).

The findings in this report are subject to at least three limitations. First, no observations of infection control and prevention practices were conducted in the dialysis center, limiting the ability to identify breaches that might have contributed to transmission. Second, the impact of residents leaving the facility for other medical appointments was not assessed. Finally, because of limited testing capacity, testing for all asymptomatic staff members in the nursing home was not performed, and records of activities for infected staff members were not available.

Effective and continual communication between dialysis centers and nursing homes is important to preventing SARS-CoV-2 transmission. If nursing homes rapidly notify dialysis centers of residents who have positive test results and those with suspected infection, dialysis centers can cohort residents (e.g., inform recommended use of personal protective equipment and provide dialysis for residents with positive test results during last shift of day with terminal cleaning) and limit exposure to others in the dialysis center (*9*). Likewise, if dialysis centers notify the nursing home in a timely manner of any community dialysis patients or dialysis staff members who had positive test results, nursing homes can perform facility-wide testing to detect asymptomatic cases and take recommended precautions (e.g., placing all exposed patients in quarantine) (*9*). Dialysis centers and nursing homes might benefit from closely reviewing the entire dialysis process, from residents leaving the facility to discharging them after dialysis, to identify practices that could contribute to SARS-CoV-2 transmission. Nursing homes might consider placing residents who undergo dialysis in single rooms close to the dialysis center with increased monitoring given their higher risk for infection. Dialysis centers and nursing homes are closely connected with a shared patient population; therefore, early identification of cases coupled with aggressive infection prevention and control actions are needed to protect medically vulnerable populations in both locations.

**Summary**

**What is already known about this topic?**

Residents of long-term care facilities have high COVID-19–associated morbidity and mortality. More information is needed about SARS-CoV-2 introduction and transmission in nursing homes.

**What is added by this report?**

Investigation of a COVID-19 outbreak in a Maryland nursing home identified a significantly higher prevalence among residents receiving dialysis (47%) than among those not receiving dialysis (16%); 72% were asymptomatic at the time of testing.

**What are the implications for public health practice?**

Nursing home residents undergoing dialysis might be at a higher risk for SARS-CoV-2 infection because of exposures to staff members and community dialysis patients. Attention to infection control practices and surveillance in nursing homes and dialysis centers is critical to preventing nursing home COVID-19 outbreaks.

## Footnotes

* These authors contributed equally to this work.

† https://coronavirus.maryland.gov/.
